# The Distinctive Forehead Cleft of the Risso’s Dolphin (*Grampus griseus*) Hardly Affects Biosonar Beam Formation

**DOI:** 10.3390/ani12243472

**Published:** 2022-12-08

**Authors:** Chong Wei, Lachlan G. Gill, Christine Erbe, Adam B. Smith, Wei-Cheng Yang

**Affiliations:** 1 Centre for Marine Science and Technology, Curtin University, Perth, WA 6102, Australia; 2 Marine Research Centre, University of Southern Denmark, 4300 Kerteminde, Denmark; 3 School of Veterinary Medicine, National Taiwan University, Taipei 10617, Taiwan

**Keywords:** Risso’s dolphin, finite element model, biosonar beam formation, CT scan, echolocation, forehead cleft

## Abstract

**Simple Summary:**

Risso’s dolphins have a sophisticated biosonar system. However, unlike other dolphins that have a round and smooth forehead, Risso’s dolphins have a distinctive vertical crease (or cleft) along the anterior surface of the forehead. Researchers have speculated how the cleft may affect biosonar beam formation given its location on the biosonar sound propagation pathway. It is almost impossible to test this experimentally. To fill this gap, this study built 2D numerical sound propagation models based on CT scans of a Risso’s dolphin. We digitally filled the cleft with neighboring soft tissues, creating a hypothetical “cleftless” head, representing a Risso’s dolphin with a round and smooth forehead as other dolphins. After comparing the sound propagation process through the original head and cleftless head, we found that the cleft played an insignificant role in forehead sound propagation and far-field beam formation. Moreover, the cleft was not responsible for the bimodal click spectrum that has previously been reported from this species. Our study presents a promising approach to advance our understanding of the function of the internal biological structures in biosonar beam formation, specifically in the absence of experimental methods to measure tissue functions directly in situ.

**Abstract:**

The Risso’s dolphin (*Grampus griseus*) has a distinctive vertical crease (or cleft) along the anterior surface of the forehead. Previous studies have speculated that the cleft may contribute to biosonar beam formation. To explore this, we constructed 2D finite element models based on computer tomography data of the head of a naturally deceased Risso’s dolphin. The simulated acoustic near-field signals, far-field signals, and transmission beam patterns were compared to corresponding measurements from a live, echolocating Risso’s dolphin. To investigate the effect of the cleft, we filled the cleft with neighboring soft tissues in our model, creating a hypothetical “cleftless” forehead, as found in other odontocetes. We compared the acoustic pressure field and the beam pattern between the clefted and cleftless cases. Our results suggest that the cleft plays an insignificant role in forehead biosonar sound propagation and far-field beam formation. Furthermore, the cleft was not responsible for the bimodal click spectrum recorded and reported from this species.

## 1. Introduction

The Risso’s dolphin (*Grampus griseus*) is an odontocete (toothed whale) species occurring world-wide in temperate and tropical waters [1]. Similar to other odontocetes, Risso’s dolphins possess a biological sonar system (biosonar) for foraging, orientation, and navigation [2,3,4]. Their echolocation clicks are broadband, with a duration of ~40–70 μs [2,3,4]. The literature suggests that Risso’s dolphins can adjust the spectral characteristics of their clicks. While most odontocetes produce clicks that are unimodal on-axis (i.e., clicks have a single peak in the frequency spectrum), Risso’s dolphins also produce bimodal clicks (exhibiting two distinct peaks in the frequency spectrum) [2,4].

**Figure 1:**
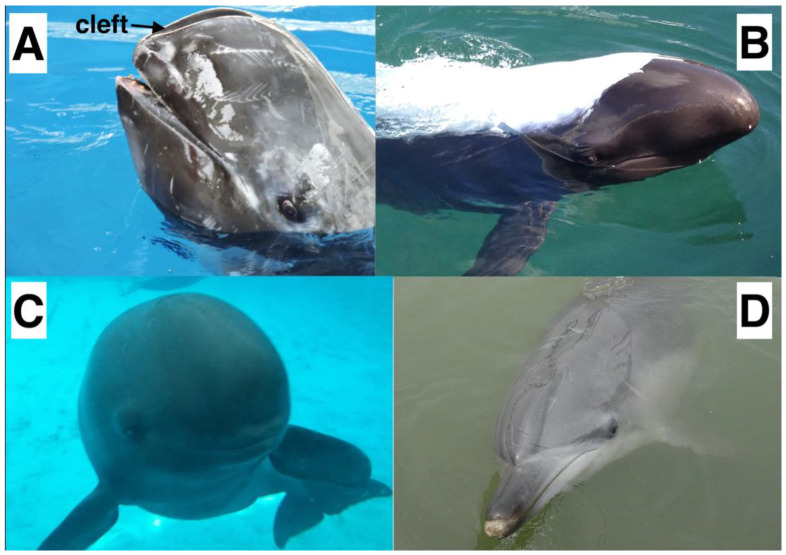
Comparison of the forehead shape between four odontocete species. (**A**) Risso’s dolphin, (**B**) false killer whale (*Pseudorca crassidens*), (**C**) Yangtze finless porpoise (*Neophocaena asiaeorientalis*), (**D**) Atlantic bottlenose dolphin.

Risso’s dolphins are morphologically different from other dolphins in that their foreheads feature a distinctive vertical crease, or a cleft, along the anterior surface of the forehead (Figure 1A). This unique cleft extends from the top of the forehead down to the tip of the rostrum [5]. It is thus located in the very region where most odontocetes project their biosonar beams [6,7,8]. The forehead is composed of various anatomical structures (e.g., the melon and connective tissues) and plays a critical role in echolocation, since it forms a pathway for sound propagation from the phonic lips (i.e., ellipsoid fatty dorsal bursae located near the posterodorsal terminus of the melon) to the surrounding water [6,9]. The role of the forehead structures has been investigated in several odontocete species, including the short-beaked common dolphin (*Delphinus delphis*) [10], bottlenose dolphin (*Tursiops truncatus*) [8,11,12,13], harbor porpoise (*Phocoena phocoena*) [7,14], and baiji (*Lipotes vexillifer*) [15]. With a gradient in acoustic impedance formed by the fatty melon (acoustic impedance increases from inner core to outer layer) and connective tissues [16], the forehead structures act as an acoustic waveguide and a collimator to channel the sound. They also provide impedance matching to transfer the acoustic energy between the animal forehead tissues and surrounding water [6,11,13,14]. However, all those odontocete species, whose biosonar click production and propagation have been studied, have a rounded and smooth forehead without a cleft. Given this unique morphological feature, previous authors speculated that the cleft might be responsible for the different spectral features seen in Risso’s dolphin clicks [2,4]. The only way to test this hypothesis is through modeling, whereby a biosonar sound propagation model be created for the clefted (original head) case, validated with field recordings of clicks, and then compared to a model of a hypothetical cleftless case.

Finite-element (FE) models have been widely used to investigate the mechanisms involved in sound production, propagation, and reception in a number of animal groups, including odontocete species [10,11,13,14,15,17,18,19,20], bats [21], and fish [22,23,24]. These models are typically based on computed tomography (CT) images of the anatomical structures. In the model, the anatomy of the animal’s head can be manipulated, such as adding or removing structures, to examine their effect on the acoustic process and determine the role of the structure in question. For example, to further investigate the role of the melon in biosonar click propagation, Wei et al. [14] constructed two FE models. The “original head” model contained all the anatomical structures seen in the CT data. Whereas in the hypothetical “no-melon” model, the inhomogeneous melon was replaced by a homogeneous one using the acoustic properties of neighboring soft tissues, and the rest of the structures remained the same. Wei et al. [14] simulated the sound propagation process frame by frame. The sound pressure field was compared between the “original head” and “no-melon” models at four reference points: where the sound waves entered the melon, one-third through the melon, half-way through the melon, and where the waves left the melon. The specific changes in beam patterns (e.g., 3-dB beamwidth and the elevation of the beam) were also determined. Wei et al. [14] concluded that the melon functioned mainly as an acoustic waveguide to direct the sound waves as they propagated through the forehead. In this study, we used a similar approach to create a hypothetical “cleftless head” model and study the possible acoustic function of the unique cleft in the head of the Risso’s dolphin based on a comparison (e.g., sound propagation, beam pattern) between the “original head” versus “cleftless head”.

## 2. Materials and Methods

### 2.1. Medical CT Scan and Image Analysis

An accurate FE model requires high-accuracy inputs of geometric information and material properties. Medical CT scanning is a non-destructive technology that provides high-resolution 3D geometry information. It allows us to study the anatomy of the interior structures of animal specimens without having to cut the samples.

**Figure 2:**
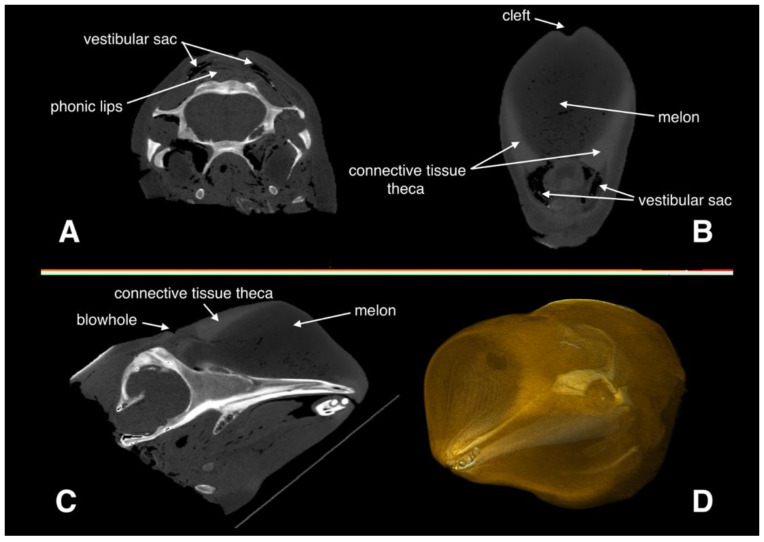
CT scan of the Risso’s dolphin’s head. (**A**) Cross plane, (**B**) frontal plane, and (**C**) sagittal plane of the head. The gray level represents the different HU values. (**D**) 3D reconstruction of the head. The yellow transparent parts are soft tissue.

A CT scan was obtained before necropsy using a mature, 2.8 m male Risso’s dolphin, which was found stranded alive, yet died after rehabilitation. The scan was completed using a 64-section multidetector CT unit (LightSpeed VCT, GE Healthcare). Images were acquired in the transaxial plane (i.e., at right angles to the long axis of the body) and helically by rotating an X-ray source of 120 kV at 320 mA. A total of 800 transverse slices of 0.625 mm thickness were collected, with a matrix size of 512 × 512 and a field of view of 30 cm × 30 cm. These parameters yielded voxel dimensions of 0.9 mm × 0.9 mm × 3.0 mm. The images were saved as DICOM files, which were later imported into the software Horos™ (Horos Project, Geneva, Switzerland) for CT data analysis. Because of gravity, the head was slightly deformed when it was scanned in the prone position. Therefore, we firstly performed 3D multiplanar reformation to adjust the positions of the head in three views so the geometry would more closely represent that of an echolocating dolphin, as shown in Figure 1A–C. The 3D geometrical model was reconstructed (Figure 2D) and the Hounsfield Unit (HU) values of each structure in the animal’s head were derived.

### 2.2. Reconstruction of Acoustic Properties

While we were unable to measure the acoustic properties of the head tissues at the time, previous studies have found linear relationships between the tissue properties (e.g., sound speed, density, and acoustic impedance) and HU values across different odontocete species [25,26,27,28]. Therefore, for our Risso’s dolphin, we relied on the HU-to-sound-speed and HU-to-density relationships from previous measurements with Yangtze finless porpoise [26]. We converted all HU measurements to sound speed and density for each structure in the head of the Risso’s dolphin, and then created 3D acoustic impedance models. More details can be found in Wei et al. [13,14], where the same approach was used to construct FE sound propagation models for a harbor porpoise and a bottlenose dolphin, respectively. The finite element analysis (FEA) results of the two species matched direct measurements from live, echolocating animals [13,14,20], proving the reliability of this approach.

### 2.3. FE Model Construction

**Figure 3:**
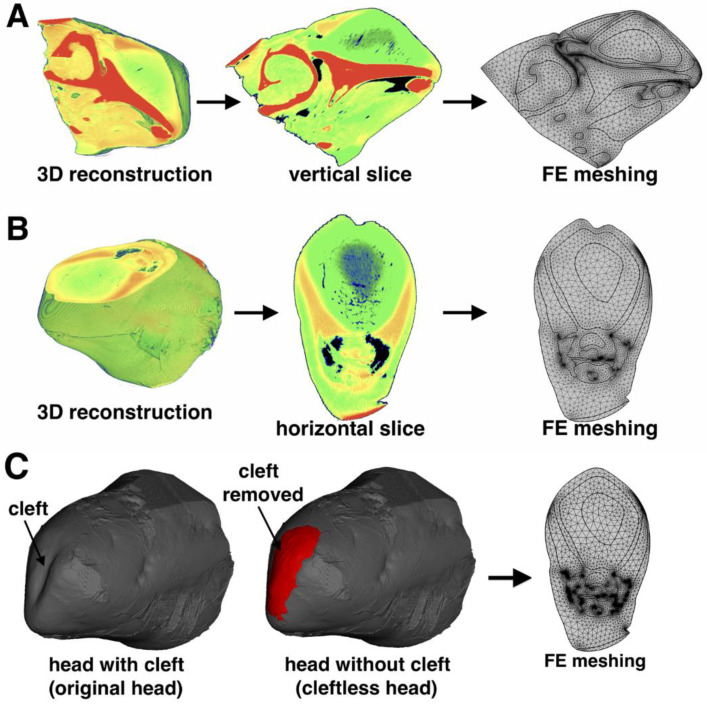
Geometric reconstruction of 2D models. (**A**) Vertical model reconstruction; the vertical slice cuts through the right phonic lips; colors represent the different acoustic impedance values. (**B**) Horizontal model reconstruction; the horizontal slice cuts through the two phonic lips and was on the major axis of the animal’s biosonar beam. (**C**) Reconstruction of the “cleftless” model. Filling of the cleft in red. For display purposes, the mesh layouts are illuminated in reduced resolution.

We selected two 2D slices from the 3D acoustic impedance model. One was a sagittal slice closest to the midline that cut through the right phonic lips (Figure 3A), which was used to create the FE model in the vertical plane. The other was a frontal slice that cut through both phonic lips on the major axis of the animal’s biosonar beam (Figure 3B), which was used to create the FE model in the horizontal plane. The two slices were imported to COMSOL Multiphysics modeling software (version 6.0; Stockholm, Sweden) for FEA and data analysis. The vertical FE model contained structures such as the right side of the phonic lips, air sacs (e.g., vestibular sac), melon, connective tissue, musculature, blubber, brain, mandibular fat, maxilla, and mandible. Besides the air sacs and a small portion of the cranium, the main structures contained in the horizontal model were soft tissues, including a pair of phonic lips (both on the left and right sides), melon, connective tissues, and musculature. As the cleft could only be observed in the horizontal model, we created a hypothetical “cleftless” model only in the horizontal plane, based on the previous horizontal model (also called the “original head/clefted” model) by filling the cleft with a structure of the same properties as the adjacent tissue (Figure 3C). The shape of the “cleftless” forehead was rounded and smooth, similar to that of other odontocete species. The sound speed and density of the structures in both models were based on the acoustic property reconstructions (Table 1).

We used COMSOL’s free mesher (Stockholm, Sweden) to generate the second-order triangular elements for mapping the entire model region; the second-order triangular elements provide a better representation of the underlying geometry in 2D models. The meshing layouts are shown in Figure 3. Mesh refinement analysis identified the optimal element size for the models as at least eight elements per wavelength λ of the center frequency $f_{c}$ of the excitation signal at the source ($\lambda =c_{water}/f_{c}$ , where $c_{water}$ is the sound speed of seawater). We also used a low-reflecting boundary condition [29] in the far-field to simulate the dolphin echolocating into an infinite seawater space.

To closely mimic the echolocation process [6], the FE computation was done in the transient time domain. The time steps of the models were set as 0.8 μs to ensure a very detailed temporal description of the high-frequency click propagation process [14]. The inhomogeneous acoustic wave equation describing the transient acoustic phenomena in a stationary fluid was solved at each grid point in the model:

(1) $$\frac{1}{\rho_{0}c^{2}}\frac{\partial^{2}p}{\partial t^{2}}+\nabla \cdot \left(-\frac{1}{\rho_{0}}\nabla p\right)=Q$$

where $\rho_{0}$ denotes the equilibrium density (kg/m^3^), $c_{s}$ the sound speed (m/s), $p=p\left(x,t\right)$ the sound pressure (Pa) as a function of space x (m) and time t (s), and $Q=Q\left(x,t\right)$ is the monopole source at location $x=x_{0}$:

(2) $$Q=\frac{4\pi }{\rho_{0}}S\delta \left(x-x_{0}\right)$$

We set a point source (i.e., monopole) at the right set of phonic lips since the size of the phonic lips was significantly smaller than the wavelengths [10,30]. The waveform of the source was modeled as a short-duration, broadband pulse, consisting of a dampened oscillation [20]. It simulated the instantaneous process of the right phonic lips opening and slapping back together after the airstream passed between them, causing the associated surrounding tissues to vibrate. The pulse can be written as:

(3) $$S=2\pi f_{0}tAe^{-\gamma t}$$

where γ is the damping rate (1/s), A is the pulse amplitude (N/m) and t is time (s). The center frequency $f_{0}$ was set as 60 kHz according to previous measurements [4].

### 2.4. Model Validation

The FE model was validated by comparing the simulated acoustic pressures in the near-field and the far-field of the dolphin to recordings from a live, echolocating dolphin. The acoustic pressure in the near-field was measured by placing an array with seven broadband suction cup hydrophones on the surface of the forehead. The hydrophones were arranged along the midline of the dolphin’s forehead (data provided by W. Lee). The far-field beam patterns were calculated from the modeled and measured pressures and then compared. Hydrophone arrays were used to measure the sound pressure on the forehead and in the far-field of a trained captive individual. To compare the simulated far-field transmission beam pattern with data from a live individual, we used data previously collected and published by Smith et al. [4] in which the two-dimensional beam patterns and directivity patterns were described for a single trained Risso’s dolphin in captivity. However, the linear transmission beam patterns were not explicitly described. Thus, data from Smith et al. [4] were reanalyzed in this paper to extract vertical and horizontal linear beam patterns, allowing more direct comparison to the FEA data here. The original data were recorded from a Risso’s dolphin that stationed horizontally in an underwater hoop, while its echolocation signals were recorded from a 16-element star-shaped array positioned 1.71 m in front of it. The array hydrophones were approximately 25 cm apart and arranged in a star-shaped pattern around a central hydrophone. The full diameter of the array measured 1.46 m, which resulted in an inner and outer ring of hydrophones being positioned at angular widths of 10.2° and 19.7° from the center hydrophone. To quantify the beam for individual clicks, click received levels were calculated on each hydrophone and then cubically interpolated over a 0.05 m mesh grid superimposed on the dimensions of the array. A contour (isopleth) was then drawn at 3 dB less than the peak received mean-square pressure level of the click. The vertical and horizontal beam widths of a click were taken as the height and width of the contour directly in line with the contour’s geometric center. Beam directivity was characterized by estimating the directivity index (*DI*) in both the vertical and horizontal planes. *DI* is defined and computed as 10 log_10_ of the ratio of the intensity of the directional biosonar beam to the intensity of an omnidirectional source of equal power. Since the dolphin biosonar beam may be approximated as a circular piston transducer [6], the following equation was used to calculate *DI* from the beamwidth in both vertical and horizontal planes [31,32]:

(4) DI=10log100.509πsinθbw22

where $\theta_{bw}$ is the 3-dB beamwidth. More details of the experimental setup and procedure for recording the far-field transmission beam can be found in Smith et al. [4].

## 3. Results

### 3.1. Model Validation

**Figure 4:**
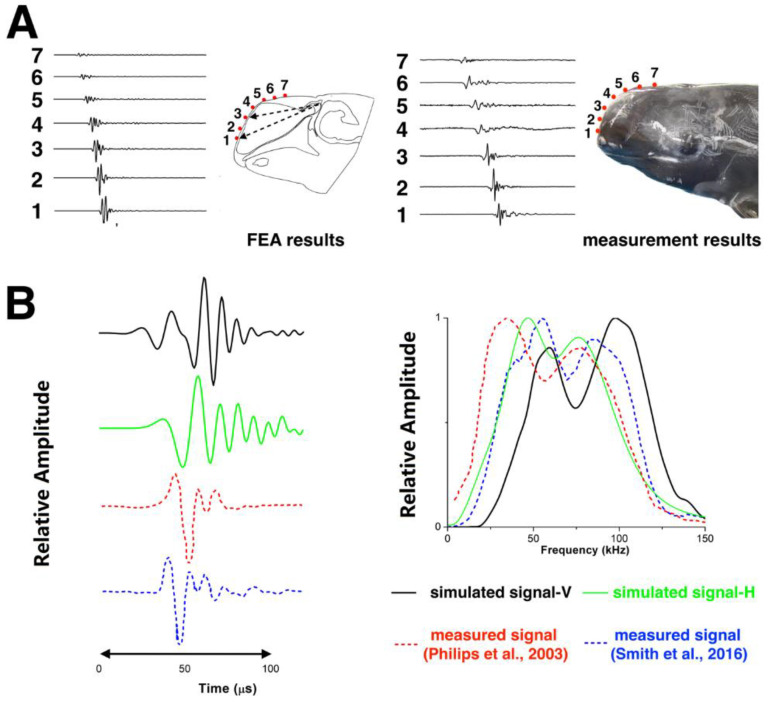
Comparison of the simulated FEA results with recordings from live, echolocating Risso’s dolphins. (**A**) Acoustic field at seven receiver points on the dolphin’s forehead: FEA results (**left**) vs. live measurements (**right**). Amplitudes of all waveforms scaled relative to the highest amplitude. (**B**) Comparison of the simulated and measured [2,4] far-field waveforms (**left**) and spectra (**right**); V: vertical model; H: horizontal model.

Figure 4A shows comparison of the modeled acoustic field on the Risso’s dolphin’s forehead in the vertical plane to the acoustic signals measured from the live dolphin at the surface of the forehead. The signal that travelled from the phonic lips through the forehead was compared at seven receiver points along the dolphin’s forehead. The points for FEA were in the same locations as the suction cup hydrophones during the live recordings. The amplitudes of the transmitted signals were relative to the highest amplitude at point 2. The relative amplitudes at points 1–3 were greater than those at points 4–7, suggesting the main beam axis was projected within the region between the two dashed arrows. The relative amplitudes of the FEA matched Lee’s measurements. The main beam axis was roughly parallel to the mandible, suggesting the outgoing beam was not oriented at a downward angle from the mandible as previously hypothesized by Philips et al. [2]. The reference plane differed between their study and ours, with Philips et al. [2] not accounting for the head angle; instead, beam properties were measured using a horizontal biteplate ensuring the animal’s mandible was parallel to the water surface.

**Figure 5:**
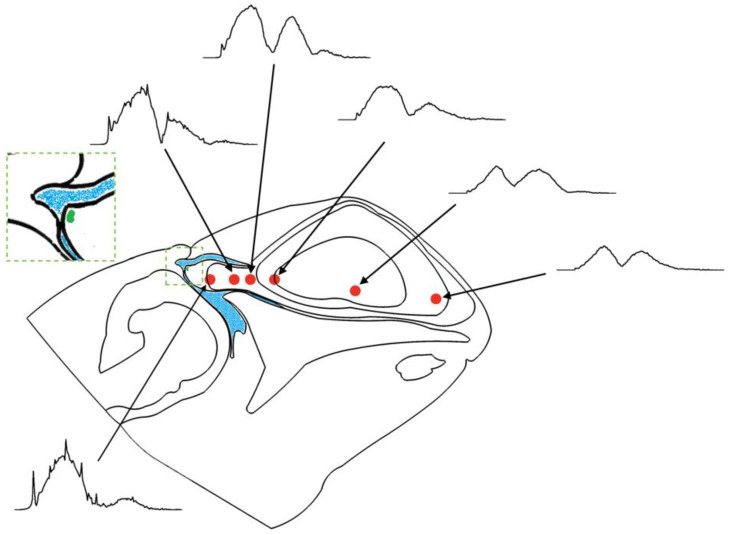
Sketch of the changes in the spectra of the transmitted signals along the propagation pathway inside the Risso’s dolphin’s head in the vertical plane. The red dots indicate receiver positions on the sound propagation path. The location of the sound source (phonic lips) is in green. The air sacs are visible in blue.

The waveforms and spectra of the simulated clicks from the vertical and horizontal models were plotted and displayed in Figure 4B, and compared to the two signals measured from live, echolocating dolphins by Philips et al. [2] and Smith et al. [4]. Modeled and measured click waveforms had the same polarity, albeit with somewhat more fluctuations at the end of the simulated horizontal click. Both the simulated clicks showed broadband click features and two prominent energy peaks in the spectra: the first peak frequencies were at 40–60 kHz and the second peak frequencies were at 75–100 kHz. Figure 5 depicts the modeled spectra at a series of receiver points in the vertical plane inside the dolphin’s head, showing how this bimodality developed.

**Figure 6:**
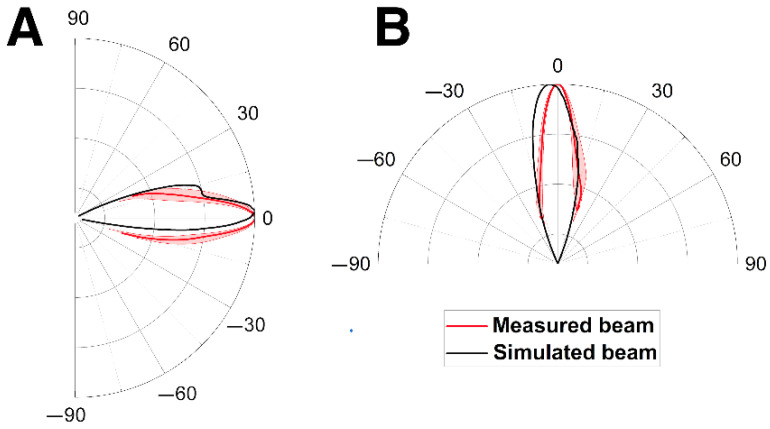
Comparison of simulated and measured far-field beam patterns in (**A**) the vertical and (**B**) the horizontal planes. The red shadow represents the standard deviation of measured data.

**Figure 7:**
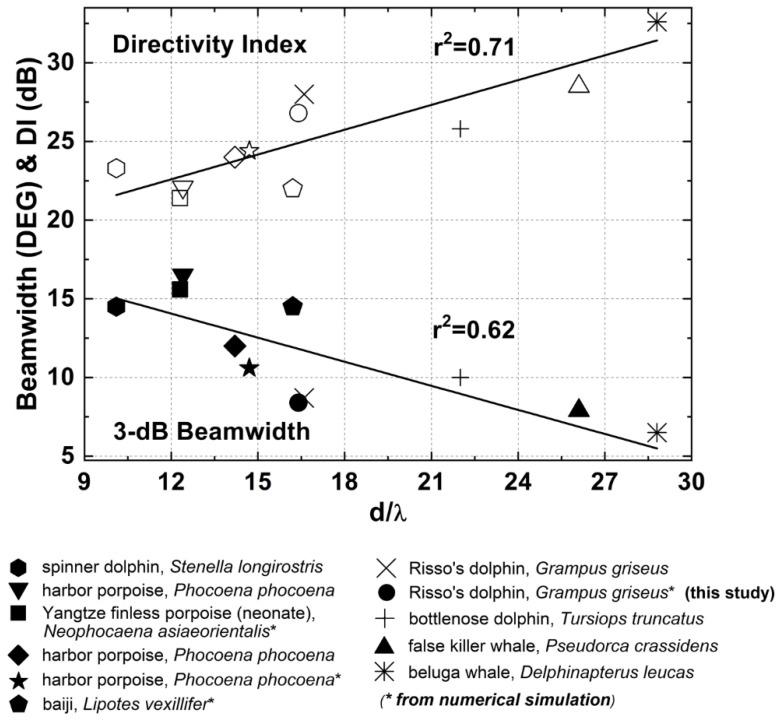
Inter-species comparison of 3-dB beamwidths and directivity indices, based on the ratio of head diameter d to the wavelength λ of the click peak frequency. The data include in situ measurements from the false killer whale [35], Risso’s dolphin [4], harbor porpoise [31,36], bottlenose dolphin [37], beluga whale (*Delphinapterus leucas*) [38], and spinner dolphin (*Stenella longirostris*) [39], as well as FEA results from the Yangtze finless porpoise [19], baiji [15], bottlenose dolphin [13], and harbor porpoise [14].

Figure 6 compares the far-field beam patterns from the vertical and horizontal models to the reanalyzed transmission beam data from Smith et al. [4], showing good agreement in shape. The elevation of the simulated beam in the vertical plane was ~2° higher than the measured one, and the simulated horizontal beam was ~5° pointed to the left compared to the measured one. Additionally, the modeled 3-dB beamwidths in the vertical and horizontal planes were 7.5° and 9.3°, respectively, compared to ~7.5° and ~6.5° measured by Smith et al. [4]. The small discrepancies could be due to the different head sizes of the individuals. Previous studies determined that the width of the beam pattern was inversely proportional to the animal’s head size [31,33,34]. The diameter at the blowhole of the scanned and modeled Risso’s dolphin in this study was ~33 cm, smaller than that of the animal measured by Smith et al. [4] (~38.2 cm), possibly explaining the observed difference (also see Figure 7).

Figure 7 compares the 3-dB beamwidths and directivity indices across different odontocete species. The ratio of the diameter *d* of the animal’s head to the wavelength *λ* of the peak frequency of its click was plotted on the x-axis. With a 33 cm diameter of the Risso’s dolphin’s head at the blowhole, a mean peak frequency of 73.5 kHz (a mean over simulated FEA results from the vertical and horizontal models), and a seawater sound speed of 1483 m/s, *d/λ* ≈ 16.4. As in previous studies [6,31], the 3-dB beamwidth was the average of the vertical and horizontal beamwidths. It was 8.4°, and so *DI* = 26.8 dB (based on Equation (4)). Both the estimated 3-dB beamwidth and the directivity index of the modeled Risso’s dolphin fitted well into the results of other odontocetes. Linear regressions across all species were strong with *r^2^* of 0.62 and 0.71, respectively, suggesting that the FE model was reliable. 

### 3.2. Sound Propagation through the Cleft

**Figure 8:**
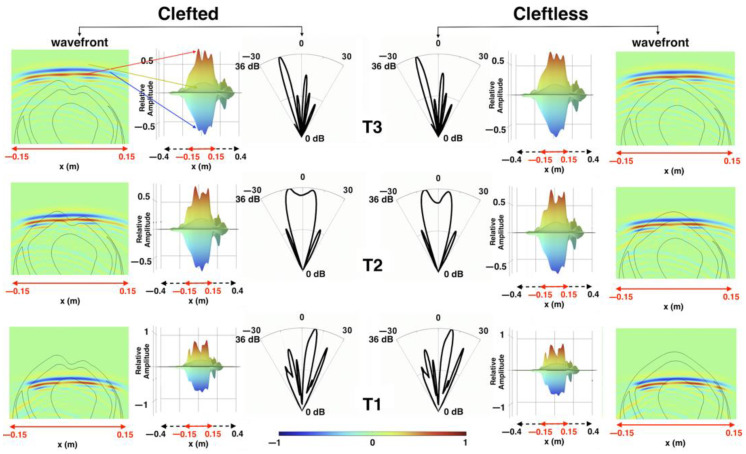
Comparison of sound propagation through the forehead cleft at three instances in time between the “clefted” case and “cleftless” case. T1: before the sound waves reached the forehead cleft, T2: when the sound waves were passing through the forehead cleft, T3: when the sound waves left the forehead cleft. The color bar shows the scale of relative sound pressure. The sound pressure was relative to the highest sound pressure value at T3. The arrows show the corresponding 3D height expression of the click wavefront. The height expression introduces 3D height to the 2D wavefront plots: the red curve of >0.5 amplitude illustrates the amplitude along the dark red wavefront in the wavefront plot (see red arrow); the blue curve with negative amplitudes corresponds to the dark blue wavefront (see blue arrow); the yellow height expression corresponds to the leading, weaker, positive wavefront (see yellow arrow).

Three instants of sound propagation through the forehead cleft (at times T1, T2, and T3) in the “clefted” versus “cleftless” cases were captured in Figure 8. Before the sound waves reached the forehead cleft (T1), the sound pressure fields and beam patterns of the two cases were identical. When the sound waves were passing through the cleft (T2), slight differences were observed at the start and end of the height expression (i.e., the amplitude along the wavefront) of the click wavefronts. The beam pattern of the cleftless case showed a lower intensity at 0°. When the sound waves left the forehead (T3), both the sound pressure field and the beam pattern were very similar in the two cases.

**Figure 9:**
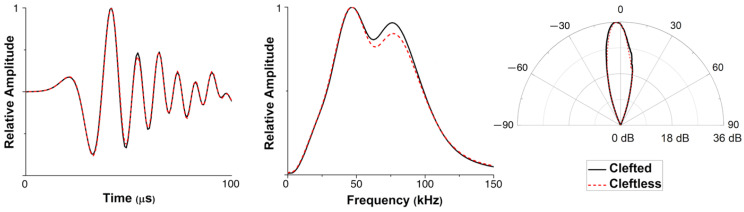
Comparison of the far-field signals, spectra, and beam patterns between the “clefted” case and “cleftless” case in the horizontal FE model.

While Figure 8 showed the similarities in the near-field, Figure 9 shows that the waveforms of the clicks transmitted to the far-field were nearly identical in both cases. The spectrum of the click in the cleftless case had slightly lower energy at the secondary peak but shared the same peak frequency at ~50 kHz. The azimuths of the main beam axes in the clefted and cleftless cases were −2.6° and −2.2°, respectively. The 3-dB horizontal beamwidth of the clefted-head beam (9.3°) was slightly wider than that of the cleftless-head beam (8.2°), suggesting the cleft might play a role in slightly widening the far-field beam.

## 4. Discussion

We developed FE models (based on CT images) to examine the potential role of the Risso’s dolphin’s forehead cleft in biosonar beam formation. We first showed that the FE model of the original (clefted) head produced clicks similar to those recorded from live, echolocating Risso’s dolphins. We then created an FE model of a hypothetical cleftless Risso’s dolphin (by filling the cleft with head tissue) to show the limited role the cleft might play in echolocation.

The FE model of the clefted Risso’s dolphin modeled clicks that were similar in waveform and spectrum in near- and far-fields to those measured from live dolphins. Modeled clicks further agreed with measured clicks in terms of their far-field beam patterns. While both modeled and measured clicks exhibited bimodal spectra, there was some variation in the peak frequencies of the lower- and higher-frequency peaks. The measured frequencies of the two energy peaks even differed across experimental studies [2,3,4]. Philips et al. [2] reported peak frequencies of the lower peak at ~30–50 kHz and of the higher peak at ~80–100 kHz. Smith et al. [4] reported peaks at ~38 and ~60 kHz. The two energy peaks of our simulated clicks appeared at 40–60 kHz and 75–100 kHz (Figure 4B). This variability may be due to individual differences but also to dynamic sound production capabilities of the dolphin [3]. The signal characteristics (e.g., spectral content) may be altered with individual and task, which is likely accomplished through manipulation of the air sacs and muscular control of the forehead structures (e.g., melon and connective tissue) [40,41]. Our FE models were constructed based on CT scans and the Risso’s dolphin specimen was static during scanning, so the FEA results could not capture the click dynamics of a dolphin echolocating under water. Therefore, our far-field transmitted click comparison in Figure 4B was qualitative rather than quantitative. Our simulated vertical click had the peak frequency at the secondary energy peak. Philips et al. [2] demonstrated that the peak frequency could appear at either the lower-frequency peak or the higher-frequency peak by showing several types of clicks from their recordings (another evidence of the dynamic nature of dolphin sound production). It should be noted that there were differences between the simulated vertical and horizontal far-field clicks (Figure 4B). These could be a result of the different positions of the two receiving points on the two different slices. Although the two points were both within the main beam axis, their positions were not exactly the same. In addition, the anatomical structures along the sound propagation pathways in the vertical and horizontal planes were significantly different. For example, there was no bony structure along the sound propagation pathway in the horizontal model, yielding different reflective components to the resultant signal in the far-field.

Based on the above results (Figure 6 and Figure 7), it was evident that the unique cleft on the forehead of the Risso’s dolphin played a limited role in the biosonar beam formation. The changes in the click wavefront and beam pattern as the sound waves travelled through the cleft between the clefted head and cleftless cases were almost undetectable. Only a slightly shifted primary projection axis (by 0.4°), a slightly widened beamwidth (by 1.1°), and a lower-energy secondary peak in the spectra were observed. Additionally, we showed that the cleft was not responsible for the previously measured and reported bimodality of the click spectra. From the FEA results, the bimodal spectrum was still present after the cleft had been filled with head tissue (Figure 7). Bimodality was also clearly visible in the spectra of the vertical clicks (Figure 3B), even though the cleft was not included in the vertical model (Figure 3A). Figure 5 showed that the bimodal spectrum was formed before the signal arrived at the forehead. Bimodality was not yet distinct when the signal had just left the phonic lips. The air sacs around the phonic lips (e.g., vestibular and premaxillary sacs) created a narrow channel for the signal to pass through—by repeated reflection, causing waveform overlays. Air sac geometry differed for every incidence, preferentially selecting different frequencies and ultimately shaping the bimodal spectrum. Therefore, the bimodal spectrum of the biosonar signal in front of the dolphin was not created by the unique, clefted shape of its forehead. One more evidence is that bimodal clicks were also found in false killer whales [35,42], which have a rounded melon like most other odontocetes (as shown in Figure 1B). It is possible that the forehead cleft serves some other not-biosonar-related biological function.

A potential limitation of our study is that we only modeled in 2D (i.e., in a vertical plane and in a horizontal plane). The cleft extends from the top of the forehead down to the tip of the rostrum. Therefore, the cleft exists in multiple 2D horizontal slices. The 2D horizontal model in this study only represented one of the slices on the main beam axis. A 3D FEA investigation would be the logical follow-on to this study to further determine the potential effects of a 3D cleft on the resultant beam.

## 5. Conclusions

In this study, we showed that the Risso’s dolphin cleft only slightly shifted the primary projection axis and mildly widened the beam, suggesting that the cleft might not play a significant role in this species’ biosonar beam formation. The results also indicated that the cleft was not responsible for the observed bimodality in the click spectra. Our study presented a promising approach to advance our understanding of the function of the internal biological structures in biosonar beam formation, specifically in the absence of experimental methods to measure tissue functions directly in situ. Our approach may benefit future biomimetic sonar technology development.

## Figures and Tables

**Table 1:** Average values of sound speed and density of the structures and seawater in the FE models.

Structures and Seawater	Sound Speed (m/s)	Density (kg/m^3^)
Melon	1378–1466	948–986
Connective tissues	1548–1581	1021–1035
Muscle	1496–1533	998–1014
Mandibular fat	1428	970
Brain	1485	994
Bony structures	3800	2000
Seawater	1483	998

## Data Availability

Not applicable.
